# The Effects of Mandatory CSR Disclosure on Tax Avoidance and Tax Incidence

**DOI:** 10.3389/fpsyg.2022.905153

**Published:** 2022-05-19

**Authors:** Rui Ding, Yuanyuan Cao, Yanqi Sun

**Affiliations:** ^1^Business School, University of Shanghai for Science and Technology, Shanghai, China; ^2^School of Economics and Management, Beijing Institute of Petrochemical Technology, Beijing, China

**Keywords:** mandatory CSR disclosure, tax avoidance, tax incidence, tax planning activities, costs of stakeholders

## Abstract

The implementation of China’s mandatory corporate social responsibility (CSR) disclosure in 2008 provides us with a natural experiment setting. In this paper, we examine the effects of mandatory CSR disclosure on the levels of firms’ tax avoidance and tax incidence. By using a difference-in-differences model, we predict and find that mandatory CSR reporting firms tend to be less tax aggressive. Then we test who bears the burden of the effective tax rate increase. It shows that the increase of effective tax rate causes a drop in firm output and imposes a tax burden on the firms’ consumers. The reduction in output also reduces demand for the firms’ inputs and after-tax returns, passing tax burden to suppliers, other stakeholders, employees, and shareholders. In contrast, there is no evidence that the decrease of firms’ tax avoidance activities influences the tax incidence of governments, banks, and other creditors. These findings provide evidence that mandatory CSR disclosure changes firm tax planning activities and indeed influences the costs of various stakeholders.

## Introduction

The appeal of coordinated development of the economy with the environment has penetrated into corporate strategies. More and more firms are willing to invest more in corporate social responsibility activities which exceed the legal standards or regulatory requirements and disclosure to the public (Kitzmueller and Shimshack, 2012). We test the effects of China’s mandatory corporate social responsibility (CSR) disclosure which was enacted at the end of 2008. The increased transparency resulting from the mandatory disclosure regime provides easier access to CSR information for regulators, investors, and stakeholders, which could also eliminate managers from extracting rents for private interests.

The implementation of China’s mandatory CSR disclosure since 2008 offers us a perfect quasi-natural experiment setting. To assess the effects of the CSR mandatory disclosure, we focus on the relationship between mandatory CSR disclosure, tax avoidance, and tax incidence. We restrict our samples of A-share firms listed on the Main Board from 2002 to 2017 and classify the treatment and control group based on whether a firm is mandatorily required to disclose a CSR report in the current year. By using a difference-in-differences (DiD) method, we first make a comparison of the change in tax avoidance level between mandatory CSR reporting firms and non-CSR reporting firms. It shows that firms tend to be less tax aggressive after the implementation of the mandatory CSR disclosure. Then we examine the tax incidence of each kind of stakeholder due to the decrease in tax avoidance after the implementation of mandatory CSR disclosure. Our results indicate that consumers, suppliers, employees, and other stakeholders bear the costs of the decrease in tax avoidance, while the tax incidence of governments, banks, and shareholders does not change.

The remainder of our paper is organized as follows. We first analyze the institutional background and develop the hypotheses, and then we describe the methodology which used in this article in section “Methodology.” Section “Results” discusses the empirical results and the last section concludes the study.

## Literature Review and Hypothesis Development

### Institutional Background

In the year of 2004, the Communist Party of China promoted a long-term goal of building a harmonious society. Since then, the Chinese government has launched a series of rules and regulations to standardize the operating activities of the firms. In December 2008, the Shanghai Stock Exchange required that firms comprise the “Corporate Governance Index,” firms with overseas-listed shares, and financial firms to disclose their CSR reports along with their annual reports since the end of 2008. Later, the Shenzhen Stock Exchange implemented a similar regulation requiring firms listed in the “Shenzhen 100 Index” to disclose their CSR reports. The announcement also emphasized that those firms which failed to disclose their CSR reports would be delisted.

### Mandatory Corporate Social Responsibility Disclosure and Tax Avoidance

Extant literature explored the relation between CSR and tax avoidance are mixed. Hoi et al. (2013) viewed CSR as part of corporate culture and documented that firms with excessive irresponsible CSR activities tend to be more tax aggressive. In contrast, Davis et al. (2016) provided evidence that corporate social responsibilities and tax avoidance are substitutes rather than complements of each other. However, studies mentioned above are processed in a setting of voluntary CSR disclosure.

Under the regime of mandatory CSR disclosure, firms need to disclose a series of tax relating data to stakeholders including the number of tax expenditures, economic performance from KPI, anti-poverty expenditures, tax revenues contributing to the local government, donations, and so on. Therefore, CSR reports provide outside stakeholders with a more transparent information environment which could also increase analyst forecast accuracy (Dhaliwal et al., 2012). Firms can also benefit from a lower cost of equity, more institutional investment, and more analyst following if they choose to issue CSR reports (Dhaliwal et al., 2011). To sum up, mandatory issued CSR reports act as supplementaries of financial reports, which provides stakeholders a more transparent channel to evaluate firms’ operating activities and reduces information asymmetry (Cho et al., 2013). As a result, when firms are on the list of disclosing their CSR activities, they face political and social pressure to cautiously alter their tax planning activities. We assume that the increased corporate transparency and the stricter supervision under the regime of mandatory CSR disclosure reduces firms’ level of tax avoidance and make the first hypothesis:

**Hypothesis 1 (H1)**. Firms tend to be less tax aggressive after the implementation of mandatory CSR disclosure.

### Mandatory Corporate Social Responsibility Disclosure, Tax Avoidance, and Tax Incidence

The burden of a specific tax includes two parts: statutory burden and tax burden (incidence). According to economics theory, we define tax incidence as the distribution effect of a specific tax on economic welfare. In other words, tax incidence means the ultimate group that bears the costs or reaps the benefits of the tax. Therefore, a change in the transaction price or transaction quantity or both will influence the tax incidence. The increase of corporate income tax, take consumers for an example, results in a higher transaction price or lower transaction volume, or both. Any changes mentioned above will impair the welfare of consumers. As a legal structure that organizes consumers, suppliers, employees, and shareholders together, a change of the level of firms’ tax avoidance will influence these stakeholders’ tax burden (Hassett and Mathur, 2015).

Under the regime of CSR disclosure, firms need to integrate legal, ethical, and philanthropic responsibilities into maximizing profit. In other words, firms should trade-off interests of each kind of stakeholder, respectively, when making decisions (Carroll, 1979). In turn, CSR activities could also benefit firms a lot, including increased employee satisfaction, higher firm value, more harmonious growth, higher investment efficiency, and lower level of information asymmetry (Edmans, 2011; Cho et al., 2013; Servaes and Tamayo, 2013; Benlemlih and Bitar, 2018).

Existing studies which examined the incidence consequences of the corporate income tax found mixed results. Harberger (1962) argued that income tax only strikes the earnings of capital, in other words, shareholders bear the tax incidence by themselves. Recent studies suggested that employees could also bear the corporate income tax incidence (Arulampalam et al., 2012; Azémar and Hubbard, 2015). In this study, we want to further explore who bears the costs or reaps the benefits of the change in tax avoidance after the implementation of mandatory CSR disclosure.

Prior literature uses macro data such as capital equipment goods prices, retail price of diesel fuel, and so on to measure tax incidence (Goolsbee, 1998; Kopczuk et al., 2013; Fuest et al., 2018). Unlike prior studies, we follow Han et al. (2016) and capture the welfare of various kinds of stakeholders by using data from firm-level cash flow statements. If the decrease in tax avoidance after the implementation of mandatory CSR disclosure correlates to changes on a firm’s specific accounts of the cash flow statement, it implies that the distribution of welfare is affected.

Our next hypotheses focus on how the incidence of various stakeholders changes along with the decrease in tax avoidance subsequent to CSR mandatory disclosure. First, we take into consideration the incidence borne by consumers. A great deal of literature tests the tax incidence subsequent to changes of different kinds of taxes. Fullerton and Metcalf (2002) reviewed existing models which explore tax incidence. For example, Mieszkowski (1972) used the general equilibrium model and found that consumers bear most of the tax incidence, while Pechman and Okner (1974) discovered that the employer share of the payroll tax is sometimes equally borne by employees and consumers. In response to the change of consumption tax, Chiou and Muehlegger (2014) proved that consumers may substitute between different tiers of cigarettes dynamically. A further study by Rozema (2018) revealed that compared with upstream firms, downstream firms pass through more consumption tax incidence to consumers. By using Consumer Expenditure data, West and Williams (2004) proved that the increase in the gasoline tax influences the consumer surplus. Marion and Muehlegger (2011) discovered a similar phenomenon that gasoline tax is passed through to consumers which are reflected in higher retail price. Concerning corporate income tax, Hassett and Mathur (2015) used consumer price level in a spatial model and found that consumers bear a small proportion of tax incidence. Furthermore, the corporate tax cut could influence consumer welfare *via* the labor supply effect (Murphy, 2016). According to the labor supply effect, a corporate tax cut reduces the disincentive effect of taxes on the labor supply, which lifts consumer welfare through a lower consumption tax burden. Thus, we assume that firms might pass part of the corporate tax burden to consumers in the form of reduced output or higher prices. This leads to the following hypothesis:

**Hypothesis 2a (H2a)**. Consumers bear the incidence of the decrease in tax avoidance due to the implementation of mandatory CSR disclosure.

Following our predictions regarding consumers, suppliers without the ability to adjust in response to price increases tend to bear tax incidence (Kotlikoff and Summers, 1986). Fankhauser and Martin (2010) found that suppliers of emissions credits bear the most incidence of a levy in developing countries without supplementarity limits. Recent studies also examined how firms pass through their tax incidence to other stakeholders including suppliers. For example, Kopczuk et al. (2013) discovered that firms pass the carbon tax incidence to their suppliers and users through the supply chain. Nerudová and Dobranschi (2016) proved that the Pigovian tax result in an increase in suppliers’ surplus at the expense of consumers’ utilities. We assume that the increase of effective tax rate will decrease their demand for supplies from upstream firms. Therefore, suppliers might bear the tax incidence because of firms’ reduced demand which leads to the following hypothesis:

**Hypothesis 2b (H2b)**. Suppliers bear the incidence of the decrease in tax avoidance due to the implementation of mandatory CSR disclosure.

Taxing jurisdictions use the tax revenue collected from firms to provide public goods, thus they are not only one of the largest of minor shareholders but also stakeholders in a firm. Existing literature proved that firms trade-off benefits between costs and try to achieve an optimal level of tax avoidance. If firms deviate from the optimal level of tax avoidance, they will quickly adjust themselves (Kim et al., 2019). The increase in the effective tax rate of corporate income tax means firms need to pay more on this tax item which disequilibrates firms’ overall optimal levels of tax avoidance. Under this circumstance, we assume that firms tend to cut expenditures on other tax items in order to converge to the optimal level. Therefore, we assume that firms are inclined to maintain a stable level of all tax expenses which leads to H2c:

**Hypothesis 2c (H2c)**. Governments do not bear the incidence of the decrease in tax avoidance due to the implementation of mandatory CSR disclosure.

A similar rationale could apply to banks and other creditors. By using aggregate data, Albertazzi and Gambacorta (2010) found that banks’ activities are influenced by the corporate income tax. Banks pass through their tax burden to their consumers at a higher price of banking services (Chiorazzo and Milani, 2011). In return, we suppose that a higher effective tax rate will reduce firms’ demand for investment, thus a downward trend of the borrowings from the bank and other creditors. On the other hand, the increase in effective tax rate aggravates firms’ tax liabilities which could increase the cost of debt. We predict that influences of these two channels might offset and make the following hypothesis:

**Hypothesis 2d (H2d)**. Banks and other creditors do not bear the incidence of the decrease in tax avoidance due to the implementation of mandatory CSR disclosure.

Concerning other stakeholders, extant studies have verified their incidence. For example, Blake (1979) made a theoretical analysis and concluded that landowners bear the whole incidence of the property tax. Similarly, French physiocrats argued that the incidence of all taxes is shifted to landowners at the expense of the rent (Basov and Bhatti, 2016). By using data on housing rates, Suárez Serrato and Zidar (2016) discovered that landowners bear 25–30 percent of tax incidence through higher rental prices because of the state corporate tax cut. We assume that the increase of effective tax rate will decrease firms’ investment and demand for supplies of products and services. Therefore, other stakeholders (landowners, advertisers, transport firms, etc.) might bear the tax incidence because of the reduced price and the quantity sold which leads to the following hypothesis:

**Hypothesis 2e (H2e)**. Other stakeholders bear the incidence of the decrease in tax avoidance due to the implementation of mandatory CSR disclosure.

Labor productivity and wages decline because tax reduces investment, therefore employees bear the tax burden (Fullerton and Heutel, 2007). Using a specific model, several studies estimated workers’ share of tax incidence. For example, Desai et al. (2007) predicted a share of 45–75 percent on labor while Arulampalam et al. (2012) predicted a share of 49 percent. Hassett and Mathur (2015) used a spatial model and find that workers bear a large proportion of the corporate tax burden through lower wages. Especially, in an open economy, employees may bear more than 100% of the corporate tax burden (Harberger, 2006). Other literature also verified that firms might pass part of the corporate tax burden to workers in the form of lower wages (Suárez Serrato and Zidar, 2016; Fuest et al., 2018). When corporate income tax cuts, firms’ tax liabilities are released which could result in a lower cost of capital so that firms need more employees and pay higher wages (Suárez Serrato and Zidar, 2016). In contrast, we infer that the decrease in tax avoidance aggravates firms’ tax liability and decreases their demand for labor. Therefore, we assume that firms shift the tax burden to employees by employing less staff and paying less on wages. Our hypothesis shows as follows:

**Hypothesis 2f (H2f)**. Employees bear the incidence of the decrease in tax avoidance due to the implementation of mandatory CSR disclosure.

Firm owners also bear a large part of the incidence under the circumstance of corporate tax cuts (Suárez Serrato and Zidar, 2016). The tax incidence of shareholders depends on the equilibrium impacts on profits. The increase of corporate income taxes reduces profits and further reduces dividends, therefore, does harm to the welfare of shareholders. We assume that current shareholders bear the incidence because of the lower after-tax returns. Our next hypothesis proceeds as follows:

**Hypothesis 2g (H2g)**. Current shareholders bear the incidence of the decrease in tax avoidance due to the implementation of mandatory CSR disclosure.

## Methodology

### Sample Selection

The implementation of mandatory CSR disclosure in 2008 provides a quasi-experiment for us to examine the correlation between mandatory CSR disclosure and tax avoidance. Since the list of firms required to disclose their CSR reports changes each year, we use a multi-period difference-in-difference (DiD) research method to compare the change of firms’ tax avoidance level between mandatory CSR reporting firms and non-CSR reporting firms. Our sample consists of 23,317 firm-year observations listed on the Shanghai Stock Exchange (SSE) and Shenzhen Stock Exchange (SZSE) between 2002 and 2017. We first exclude firms listed in the Growth Enterprise Board because the preferential tax policies of these firms are a little different from that of the Main Board, and we also exclude firms in the financial industries. Then we exclude firm-year observations with missing data on the dependent variable, independent variables, and control variables. To control for the unintended influence of outliers in the tests, we winsorize all the continuous variables at the top and bottom 1 percent by years.

### Measure of Tax Avoidance

*ETR* is defined as the ratio of total income tax expense paid to pre-tax profit. Considering the existence of preferential tax policies and other factors, the nominal tax rates change every year, therefore we include another measurement of tax avoidance (*NETR*). Before computing *NETR*, extreme values of *ETR*s which are above 1 (below –1) are reset to 1 (–1). Then we definite *NETR* as the ratio of *ETR* to the nominal tax rate.

### Test of Hypothesis 1

To test our first prediction about the relationship between mandatory CSR disclosure and tax avoidance, we employ an ordinary least squares (OLS) regression that adjusts standard errors for firm-level clustering and controls for the year and industry effects, as follows:


$$T\,A=\beta_{0}+\beta_{1}\,M\,A\,N\,D\,A\,T\,O\,R\,Y+\Sigma \,\beta_{k}\,C\,o\,n\,t\,r\,o\,l\,s_{i\,t}$$

(1) +Y⁢e⁢a⁢r&I⁢n⁢d⁢u⁢s⁢t⁢r⁢y⁢F⁢E+ε

We use two indexes to measure firms’ tax avoidance level (*TA*): *ETR* and *NETR*. We divide the full samples into mandatory CSR reporting firms and non-CSR reporting firms. To test our first hypothesis, we examine β_*1*_, the coefficient on *MANDATORY*, which represents the effect of mandatory CSR disclosure on firms’ tax avoidance level. A positive coefficient on *MANDATORY* would reveal that firms tend to be less tax aggressive subsequent to the implementation of mandatory CSR disclosure, thus we predict that β_1_ > 0.

Those firm characteristics mentioned in the prior studies which are correlated with tax avoidance are controlled (Dyreng et al., 2008; Frank et al., 2009; Chen et al., 2010). We first include the set of *ROA*, *SALES*, and *LEV* into *Controls* to control for the firms’ operating performance. *ROA* equals a firm’s earnings before interest and tax divided by assets which controls for the influence of firm profitability on tax avoidance. Then we include *SALES* and *LEV*, which equals the natural log of a firm’s sales and the ratio of long-term debt to total assets to control the effects of operating income and leverage on tax avoidance. The second set of *Controls* consists of *PPE*, *INTANG*, and *INVENTORY*, which represent the ratio of the sum of property, plant, and equipment to total assets, the ratio of the net balance of intangible assets to total assets, and the ratio of the net balance of inventories to total assets, respectively. We use these variables to control the underlying influence of book-tax differences. In addition, prior studies revealed that growing firms tend to invest more tax-preferential assets which arise timing differences when recognizing expenses (Chen et al., 2010). Therefore, we also control for firm size and growth (*SIZE* and *MB*) as well as the year and industry dummies to control the fixed effect. Detailed variable definitions are shown in Appendix Table A1.

### Test of Hypothesis 2a Through Hypothesis 2g

To test our second prediction about which stakeholder bears the costs or reap the benefits of tax avoidance level change due to the implementation of mandatory CSR disclosure, we employ a change regression specification. Such analysis of the change model helps us to eliminate unintended influence from omitted variables, which shows as follow:


$$\Delta \,T\,I=\beta_{0}+\beta_{1}\,\Delta \,M\,A\,N\,D\,A\,T\,O\,R\,Y+\beta_{2}\,\Delta \,I\,N\,C\,R\,E\,A\,S\,E$$

$$+\beta_{3}\,\Delta \,M\,A\,N\,D\,A\,T\,O\,R\,Y\,\_\,I\,N\,C\,R\,E\,A\,S\,E+\Sigma \,\beta_{k}\,C\,o\,n\,t\,r\,o\,l\,s_{i\,t}$$

(2) +Y⁢e⁢a⁢r&I⁢n⁢d⁢u⁢s⁢t⁢r⁢y⁢F⁢E+ε

We use *TI* to include all dependent variables which measure the tax incidence of various kinds of stakeholders (*CONSUMER*, *SUPPLIER*, *GOVERN*, *OSTAKE*, *BANK*, *EMPLOYEE*, and *SHAREHOLDER*). *CONSUMER* is defined as cash received for selling goods and providing services divided by lagged total assets. *SUPPLIER* is measured as cash paid for purchasing goods and receiving services divided by lagged total assets. *GOVERN* is the ratio of the cash payment of taxes and expenses to lagged total assets. *OSTAKE* is defined as other cash paid to relating operating activities divided by lagged total assets. *BANK* is calculated by dividing lagged total assets by the cash payment of borrowings. *EMPLOYEE* equals cash paid to and on behalf of employees divided by lagged total assets. *SHAREHOLDER* is defined as cash paid for distributing dividends, profits, and interest payment divided by lagged total assets.

In order to differentiate firms experiencing effective tax rate increases or decreases after the implementation of mandatory CSR disclosure, we separate the sample using *INCREASE1* or *INCREASE2* which is a dummy variable equals to 1 if the firm experiences an increase on *ETR* or *NETR* in the current year. We examine β_*3*_, the coefficient on intersection between *MANDATORY* and *INCREASE1* or *INCREASE2* (*MANDATORY_INCREASE1* or *MANDATORY_INCREASE2*), to figure out the effect of firms’ tax avoidance change subsequent to mandatory CSR disclosure on tax incidence of various stakeholders. All the variables with the prefix “Δ” symbol the differences in values between the current year and prior year. Detailed variable definitions are in Appendix Table A1.

## Results

### Descriptive Statistics and Correlations

To reduce the influence of dramatic market changes to the sample selection due to China’s joining WTO in 2001, we restrict the sample period spanning from 2002 to 2017. It consists of 23,317 firm-year observations for publicly traded firms with data from CSMAR. Table 1 provides detailed sample information by year and industry. Untabulated results show that the sample firms experience a decrease in the effective tax rate due to a series of tax reduction policies enacted by the Chinese government in the pre-2008 period. However, the year 2008 witnessed a turning point when the firms’ effective tax rates increase steadily, which might be due to the mandatory disclosure of CSR reports beginning from the end of 2008. Since then, about 20% of the sample firms are required to disclose their CSR reports to the public each year.

**TABLE 1:** Descriptive statistics and correlations.

Panel A: Descriptive statistics											
**Variable**	**Mean**	***SD***	**Min**	**p25**	**Median**	**p75**	**Max**				
*ETR*	0.198	0.216	–0.953	0.106	0.171	0.265	1.000
*NETR*	0.952	1.004	–4.000	0.553	0.943	1.183	6.667
*MANDATORY*	0.128	0.334	0	0	0	0	1.000
*ROA*	0.030	0.070	–0.688	0.011	0.032	0.060	0.247
*SALES*	21.118	1.546	16.320	20.146	21.037	22.010	26.207
*LEV*	0.489	0.241	0.042	0.324	0.486	0.635	3.362
*PPE*	0.263	0.182	0.001	0.121	0.231	0.379	0.807
*INTANG*	0.045	0.056	0	0.011	0.029	0.057	0.443
*INVENTORY*	0.164	0.152	0	0.063	0.125	0.210	0.796
*SIZE*	21.788	1.313	18.724	20.870	21.626	22.522	26.651
*MB*	3.829	4.881	–16.760	1.723	2.670	4.390	64.963

**Panel B: Correlation analysis**											

	**(1)**	**(2)**	**(3)**	**(4)**	**(5)**	**(6)**	**(7)**	**(8)**	**(9)**	**(10)**	**(11)**

1-ETR	–	**0.857**	**0.035**	0.006	**0.112**	**0.063**	**–0.017**	**–0.047**	**0.083**	**0.093**	**–0.117**
2-NETR	**0.907**	–	**0.034**	**0.035**	**0.083**	**–0.011**	**–0.064**	0.003	**0.090**	**0.060**	**–0.056**
3-MANDATORY	–0.008	0.005	–	**0.061**	**0.382**	**0.095**	**–0.023**	**0.018**	0.005	**0.419**	**–0.117**
4-ROA	**–0.023**	**0.018**	**0.079**	–	**0.156**	**–0.435**	**–0.103**	**–0.017**	**–0.081**	**0.068**	**0.202**
5-SALES	**–0.027**	**–0.016**	**0.418**	**0.224**	–	**0.284**	**0.039**	–0.009	**0.098**	**0.860**	**–0.256**
6-LEV	**0.073**	**0.026**	**0.064**	**–0.460**	**0.154**	–	**0.023**	**–0.070**	**0.209**	**0.291**	**–0.108**
7-PPE	**0.011**	**–0.023**	–0.009	**–0.101**	**0.057**	**0.056**	–	**0.169**	**–0.358**	–0.003	**–0.165**
8-INTANG	0.009	0.005	**–0.024**	**–0.061**	**–0.069**	0.008	**0.039**	–	**–0.147**	**–0.040**	**0.070**
9-INVENTORY	**0.070**	**0.050**	**0.039**	**–0.032**	**0.058**	**0.210**	**–0.419**	**–0.206**	–	**0.024**	0.005
10-SIZE	**–0.021**	**–0.020**	**0.487**	**0.145**	**0.871**	**0.184**	**0.042**	**–0.044**	**0.086**	–	**–0.323**
11-MB	**–0.057**	**–0.058**	**–0.077**	**0.030**	**–0.224**	0.002	**–0.103**	**0.056**	**–0.024**	**–0.243**	–

*Panel A presents descriptive statistics for the variables used in the empirical analyses. The sample consists of 23317 firm-year observations spanning from 2002 to 2017 for publicly traded firms with data from CSMAR and CCER. Measurements of tax avoidance (ETR and NETR) are constrained to lie on the [–1,1] interval. All continuous variables are winsorized at the 1st and 99th percentiles to mitigate the effect of outliers. Variable definitions in Appendix Table A1. Panel B presents correlations for our independent variables and variables of interest (ETR and NETR). Pearson correlation coefficients are above the diagonal, while Spearman correlation coefficients are below the diagonal. Bold values denote significance at the 1, 5, or 10% level. All continuous variables are winsorized at the 1st and 99th percentiles to mitigate the effect of outliers. Variable definitions in Appendix Table A1.*

Table 1 reports descriptive statistics of the variables and correlations of them. As observed from Panel A, the mean value of *ETR* is 0.198 which is much lower than the statuary tax rate (25%) and *NETR* is also smaller than 1, which reveals that tax avoidance is a common phenomenon among sample firms. The average value of *MANDATORY* is 0.128, which means only 12.8% of the observations are required to mandatary disclosure CSR reports. Panel B contains the univariate Pearson and Spearman correlations between *MANDATORY*, our variable of interest (*ETR* and *NETR*), and other control variables. The upper triangle shows Pearson correlation coefficients, while the lower triangle presents Spearman correlation coefficients. We find that the correlations between *ETR*, *NETR*, and most of the control variables are significant. Though *ETR* and *NETR* are not correlated with *MANDATORY* using the model of Spearman correlation, *ETR* and *NETR* are positively correlated with *MANDATORY* using the model of Pearson correlation, which partly proves H1.

### The Base Model Regression

In Table 2, we use equation (1) to test our first hypothesis that firms tend to be less tax aggressive subsequent to the implementation of mandatory CSR disclosure. We report empirical results and significance levels after clustering by firms. The results support H1 that *MANDATORY* is significantly positively correlated with two measures of tax avoidance: *ETR* (0.0194, *p* < 0.01) and *NETR* (0.0992, *p* < 0.01). It proves that firms experience a decrease in tax avoidance after being required to disclosure CSR reports. Among control variables, we find that firms with a higher return on assets, higher leverage, more property, plant, and equipment, more intangible assets, and more inventories tend to be less tax aggressive. In contrast, firms with larger assets and higher market-to-book ratios are inclined to pay fewer taxes.

**TABLE 2:** Test of H1.

Dependent variable	Predicted sign	(1) *ETR*	(2) *NETR*
*MANDATORY*	+	0.0194***	0.0992***
		(3.56)	(3.67)
*ROA*		0.1199***	0.8521***
		(2.89)	(4.48)
*SALES*		–0.0008	0.0063
		(–0.23)	(0.39)
*LEV*		0.0490***	0.1727**
		(3.54)	(2.48)
*PPE*		0.0310**	–0.0640
		(2.27)	(–0.99)
*INTANG*		0.1456***	0.4803***
		(3.90)	(2.78)
*INVENTORY*		0.1047***	0.3336***
		(5.27)	(3.86)
*SIZE*		–0.0066*	–0.0496***
		(–1.65)	(–2.79)
*MB*		–0.0020***	–0.0128***
		(–4.76)	(–6.83)
Cons		0.2974***	1.5823***
		(6.48)	(7.41)
Industry and Year FE		YES	YES
SE clustered by firm		YES	YES
N (firm-years)		23317	23317
Adjusted *R*^2^		0.0651	0.0323

Measurements of tax avoidance (ETR and NETR) are constrained to lie on the [–1,1] interval. We use industry-level and year-level fixed effects. Fixed effects are omitted for parsimony. Standard errors are robust to heteroscedasticity and clustered at the firm level, following the suggestions in Petersen (2009). We perform one-sided t-tests on each coefficient. T-statistics are in parentheses. ***, **, and * denote significance at the 1, 5, and 10% level, respectively.

### Tests of Hypothesis 2a Through Hypothesis 2g

Table 3 presents the results of tax incidence of each kind of stakeholder subsequent to the decrease in tax avoidance due to mandatory disclosure of CSR reports. We use the intersection between *MANDATORY* and *INCREASE1* (*INCREASE2*) to construct a firm-specific variable of the mandatory CSR disclosure’s effect on *ETR*s (*NETR*s) to assess the incidence. In this part, we focus on the coefficients on *ΔMANDATORY_INCREASE1* and *ΔMANDATORY_INCREASE2*.

**TABLE 3:** Test of H2a-H2g.

Panel A: Tests of H2a-H2b				
	**(1)** ***ΔCONSUMER***	**(2)** ***ΔCONSUMER***	**(3)** ***ΔSUPPLIER***	**(4)** ***ΔSUPPLIER***
*ΔMANDATORY*	–0.0807***	–0.0808***	–0.0516***	–0.0518***
	(–3.47)	(–3.48)	(–2.98)	(–2.97)
*ΔINCREASE1*	0.0063		0.0072	
	(1.25)		(1.57)	
*ΔMANDATORY_INCREASE1*	–0.0211*		–0.0182*	
	(–1.94)		(–1.79)	
*ΔINCREASE2*		0.0082*		0.0080*
		(1.65)		(1.76)
*ΔMANDATORY_INCREASE2*		–0.0210*		–0.0180*
		(–1.83)		(–1.69)
*ΔROA*	0.3849***	0.3824***	0.1779***	0.1766***
	(7.02)	(6.99)	(3.68)	(3.65)
*ΔSALES*	0.3825***	0.3825***	0.3053***	0.3053***
	(18.19)	(18.19)	(16.39)	(16.38)
*ΔLEV*	0.1150**	0.1145**	0.1068*	0.1066*
	(2.02)	(2.01)	(1.91)	(1.91)
*ΔPPE*	–0.1380**	–0.1372**	–0.2059***	–0.2054***
	(–2.35)	(–2.34)	(–4.25)	(–4.24)
*ΔINTANG*	0.0055	0.0048	–0.0474	–0.0482
	(0.03)	(0.03)	(–0.37)	(–0.38)
*ΔINVENTORY*	0.2736**	0.2740**	0.5828***	0.5831***
	(2.42)	(2.42)	(5.20)	(5.20)
*ΔSIZE*	0.3605***	0.3606***	0.2915***	0.2915***
	(11.35)	(11.36)	(9.68)	(9.69)
*ΔMB*	0.0020	0.0020	0.0019	0.0019
	(1.39)	(1.39)	(1.30)	(1.30)
Cons	0.0125	0.0144	0.0037	0.0052
	(0.92)	(1.04)	(0.31)	(0.43)
Industry and Year FE	YES	YES	YES	YES
N (firm-years)	19,282	19,282	19,282	19,282
Adjusted *R*^2^	0.2529	0.2530	0.2339	0.2339

**Panel B: Tests of H2c-H2d**				

	**(1)** ***ΔGOVERN***	**(2)** ***ΔGOVERN***	**(3)** ***ΔBANK***	**(4)** ***ΔBANK***

*ΔMANDATORY*	–0.0059***	–0.0060***	–0.0128	–0.0121
	(–3.25)	(–3.30)	(–1.31)	(–1.24)
*ΔINCREASE1*	0.0004		0.0017	
	(1.03)		(0.84)	
*ΔMANDATORY_INCREASE1*	–0.0007		–0.0049	
	(–0.99)		(–1.04)	
*ΔINCREASE2*		0.0004		0.0022
		(1.06)		(1.13)
*ΔMANDATORY_INCREASE2*		–0.0005		–0.0063
		(–0.74)		(–1.32)
*ΔROA*	0.0403***	0.0402***	0.0973***	0.0967***
	(7.66)	(7.66)	(3.16)	(3.14)
*ΔSALES*	0.0161***	0.0161***	0.0303***	0.0303***
	(13.00)	(13.00)	(5.51)	(5.51)
*ΔLEV*	–0.0112**	–0.0112**	–0.0290	–0.0290
	(–2.55)	(–2.55)	(–1.34)	(–1.35)
*ΔPPE*	–0.0006	–0.0006	0.0469**	0.0471**
	(–0.13)	(–0.13)	(2.01)	(2.02)
*ΔINTANG*	0.0153	0.0153	0.0750	0.0749
	(1.15)	(1.14)	(1.21)	(1.21)
*ΔINVENTORY*	0.0035	0.0035	0.0399	0.0400
	(0.48)	(0.48)	(1.20)	(1.20)
*ΔSIZE*	0.0273***	0.0273***	0.1517***	0.1518***
	(12.50)	(12.50)	(14.96)	(14.96)
*ΔMB*	0.0001	0.0001	0.0005	0.0005
	(0.92)	(0.92)	(0.93)	(0.93)
Cons	–0.0033***	–0.0032***	0.0271***	0.0276***
	(–3.00)	(–2.92)	(3.30)	(3.36)
Industry and year FE	YES	YES	YES	YES
N (firm-years)	19,282	19,282	19,282	19,282
Adjusted *R*^2^	0.1888	0.1888	0.0981	0.0982

**Panel C: Tests of H2e-H2f**				

	**(1)** ***ΔOSTAKE***	**(2)** ***ΔOSTAKE***	**(3)** ***ΔEMPLOYEE***	**(4)** ***ΔEMPLOYEE***

*ΔMANDATORY*	–0.0084***	–0.0086***	–0.0037***	–0.0037***
	(–2.70)	(–2.79)	(–2.61)	(–2.63)
*ΔINCREASE1*	0.0010		0.0005	
	(1.01)		(1.49)	
*ΔMANDATORY_INCREASE1*	–0.0032**		–0.0013**	
	(–2.26)		(–2.12)	
*ΔINCREASE2*		0.0011		0.0005
		(1.15)		(1.49)
*ΔMANDATORY_INCREASE2*		–0.0027*		–0.0012**
		(–1.94)		(–2.00)
*ΔROA*	0.0121	0.0118	0.0189***	0.0188***
	(0.86)	(0.84)	(4.06)	(4.05)
*ΔSALES*	0.0201***	0.0201***	0.0112***	0.0112***
	(5.54)	(5.54)	(9.45)	(9.45)
*ΔLEV*	0.0117	0.0116	0.0050	0.0050
	(1.00)	(0.99)	(1.05)	(1.05)
*ΔPPE*	–0.0340***	–0.0340***	0.0017	0.0018
	(–3.39)	(–3.38)	(0.38)	(0.38)
*ΔINTANG*	0.0235	0.0234	0.0266*	0.0265*
	(0.68)	(0.67)	(1.94)	(1.94)
*ΔINVENTORY*	0.0158	0.0158	–0.0047	–0.0047
	(0.75)	(0.75)	(–0.82)	(–0.82)
*ΔSIZE*	0.0584***	0.0584***	0.0253***	0.0253***
	(11.24)	(11.24)	(12.49)	(12.49)
*ΔMB*	0.0005**	0.0005**	0.0000	0.0000
	(2.19)	(2.19)	(0.30)	(0.30)
Cons	–0.0025	–0.0023	0.0029**	0.0030**
	(–0.69)	(–0.63)	(2.14)	(2.19)
Industry and year FE	YES	YES	YES	YES
N (firm-years)	19,282	19,282	19,282	19,282
Adjusted *R*^2^	0.0830	0.0829	0.1342	0.1342

**Panel D: Tests of H2g**		

	**(1)** ***ΔSHAREHOLDER***	**(2)** ***ΔSHAREHOLDER***

*ΔMANDATORY*	–0.0035***	–0.0034***
	(–2.82)	(–2.81)
*ΔINCREASE1*	0.0007***	
	(3.24)	
*ΔMANDATORY_INCREASE1*	–0.0011**	
	(–2.01)	
*ΔINCREASE2*		0.0007***
		(3.28)
*ΔMANDATORY_INCREASE2*		–0.0011**
		(–2.07)
*ΔROA*	0.0123***	0.0122***
	(3.23)	(3.21)
*ΔSALES*	0.0029***	0.0029***
	(5.21)	(5.21)
*ΔLEV*	0.0090***	0.0090***
	(3.95)	(3.95)
*ΔPPE*	0.0079***	0.0079***
	(3.03)	(3.04)
*ΔINTANG*	0.0148**	0.0147**
	(2.19)	(2.18)
*ΔINVENTORY*	0.0007	0.0007
	(0.20)	(0.20)
*ΔSIZE*	0.0178***	0.0178***
	(17.41)	(17.41)
*ΔMB*	0.0001	0.0001
	(1.36)	(1.36)
Cons	–0.0043***	–0.0042***
	(–3.88)	(–3.77)
Industry and year FE	YES	YES
SE clustered by firm	YES	YES
N (firm-years)	19,282	19,282
Adjusted *R*^2^	0.1036	0.1036		

Panel A reports the incidence of consumers and suppliers due to the increase in tax avoidance after the implementation of mandatory CSR disclosure. We use industry-level and year-level fixed effects. Standard errors are robust to heteroscedasticity and clustered at the firm level. T-statistics are in parentheses. ***, **, and * denote significance at the 1, 5, and 10% level, respectively. Panel B reports the incidence of governments, banks, and other creditors due to the increase in tax avoidance after the implementation of mandatory CSR disclosure. Standard errors are robust to heteroscedasticity and clustered at the firm level. T-statistics are in parentheses. ***, **, and * denote significance at the 1, 5, and 10% level, respectively. Panel C reports the incidence of other stakeholders and employees due to the increase in tax avoidance after the implementation of mandatory CSR disclosure. Our control variables are defined in Appendix Table A1. We use industry-level and year-level fixed effects. Standard errors are robust to heteroscedasticity and clustered at the firm level. T-statistics are in parentheses. ***, **, and * denote significance at the 1, 5, and 10% level, respectively. Panel D reports the incidence of current shareholders due to the increase in tax avoidance after the implementation of mandatory CSR disclosure. Our control variables are defined in Appendix Table A1. We use industry-level and year-level fixed effects. Standard errors are robust to heteroscedasticity and clustered at the firm level. T-statistics are in parentheses. ***, **, and * denote significance at the 1, 5, and 10% level, respectively.

Panel A presents the results of whether consumers and suppliers bear the incidence of the decrease in tax avoidance after the implementation of mandatory CSR disclosure. Consistent with H2a, the coefficients on *ΔMANDATORY_INCREASE1* and *ΔMANDATORY_INCREASE2* in columns (1 and 2) equal –0.0211 (*p* < 0.1) and –0.0210 (*p* < 0.1), respectively, which suggest that the decrease in tax avoidance after the implementation of mandatory CSR disclosure results in a reduction in cash received for selling goods and providing services. Furthermore, the negatively significant coefficients on *ΔMANDATORY_INCREASE1* and *ΔMANDATORY_INCREASE2* means that the effect of reduced output exceeds higher prices of products sold. Similarly, we find that the coefficients on *ΔMANDATORY_INCREASE1* and *ΔMANDATORY_INCREASE2* in columns (3 and 4) are also negatively significant. It shows that the decrease in tax avoidance after the implementation of mandatory CSR disclosure results in a decreased demand for supplies which verifies H2b.

In Panel B, we predict that governments, banks, and other creditors do not bear the incidence of the decrease in tax avoidance after the implementation of mandatory CSR disclosure. The coefficients on *ΔMANDATORY_INCREASE1* and *ΔMANDATORY_INCREASE2* in columns (1–4) presented are not significant, which suggests that the decrease in tax avoidance after the implementation of mandatory CSR disclosure has no effects on cash payment of taxes, expenses, and borrowings.

H2e and H2f predict that other stakeholders and employees bear the incidence of the decrease in tax avoidance due to the implementation of mandatory CSR disclosure. As reported in columns (1 and 2) in Panel C, the coefficients on intersection variables equal –0.0032 (*p* < 0.05) and –0.0027 (*p* < 0.05), which reveal that the effect of decreased demand for supplies is bigger than the higher supply prices so that leads to a negative net effect. Thus, we conclude that the decrease in tax avoidance after the implementation of mandatory CSR disclosure results in a reduction in other cash paid to relating operating activities, which proves H2e. Referring to employees, the coefficients on *ΔMANDATORY_INCREASE1* and *ΔMANDATORY_INCREASE2* in columns (3 and 4) equal –0.0013 (*p* < 0.05) and –0.0012 (*p* < 0.05), which suggest that the decrease in tax avoidance after the implementation of mandatory CSR disclosure leads to a reduction in cash paid to and on behalf of employees.

Panel D reports the results of testing H2g, which assumes that current shareholders bear the incidence of the decrease in tax avoidance after the implementation of mandatory CSR disclosure. The coefficients on *ΔMANDATORY_INCREASE1* and *ΔMANDATORY_INCREASE2* are both negative and significant at the 5% level, which supports H2g that the decrease in tax avoidance after the implementation of mandatory CSR disclosure results in a reduced cash payment paid for distributing dividends, profits, and interest payment.

### Additional Analyses

#### Alternative Measures of Tax Avoidance

To make sure that our base model regression is robust, we use alternative measures to calculate tax avoidance. *CETR* is measured as the ratio of cash payment for income tax to pre-tax profit. Considering the existence of preferential tax policies and other factors, the nominal tax rates change every year, therefore we also include another measurement of tax avoidance (*NCETR*). Prior to computing *NCETR*, extreme values of *CETR*s which are above 1 (below –1) are reset to 1 (–1). Then we definite *NCETR* as the ratio of *CETR* to the nominal tax rate. The results of these two alternative measures are presented in columns (1 and 2) of Table 4, where we observe similar main results with Table 3.

**TABLE 4:** Robustness test.

Dependent variable	(1) *CETR*	(2) *NCETR*	(3) *StdDev_ETR*	(4) *StdDev_NETR*
*MANDATORY*	0.0452***	0.3109***	–0.1787*	–0.2040**
	(3.34)	(3.63)	(–1.93)	(–2.14)
*ROA*	1.7491***	8.5920***	–8.4311***	–8.7138***
	(20.67)	(19.51)	(–13.37)	(–13.51)
*SALES*	0.0711***	0.3821***	–0.1508***	–0.1478***
	(10.75)	(10.19)	(–3.21)	(–3.13)
*LEV*	0.0545**	0.0855	0.7568***	0.7160***
	(1.99)	(0.60)	(3.92)	(3.79)
*PPE*	0.1085***	–0.2069	0.3935*	0.3597*
	(3.98)	(–1.27)	(1.92)	(1.65)
*INTANG*	0.1468**	0.0643	0.4007	0.1866
	(2.12)	(0.16)	(0.69)	(0.31)
*INVENTORY*	0.3319***	0.9856***	–0.4431*	–0.5174**
	(9.11)	(4.91)	(–1.71)	(–1.98)
*SIZE*	–0.0597***	–0.3870***	0.0189	0.0376
	(–7.49)	(–8.32)	(0.34)	(0.67)
*MB*	–0.0090***	–0.0525***	0.0334***	0.0360***
	(–9.17)	(–10.78)	(4.53)	(4.73)
Cons	0.0419	1.8169***	3.8884***	3.5102***
	(0.40)	(2.99)	(5.51)	(4.51)
Industry and year FE	YES	YES	YES	YES
SE clustered by firm	YES	YES	YES	YES
N (firm-years)	23,317	23,317	20,703	20,703
Adjusted *R*^2^	0.1290	0.1166	0.0668	0.0622

Columns (1) and (2) report alternative measurements of tax avoidance (CETR and NCETR), which are constrained to lie on the [–1,1] interval. Columns (3) and (4) report the volatility of tax avoidance (StdDev_ETR and StdDev_NETR) from 2002 to 2016. We use industry-level and year-level fixed effects. We use industry-level and year-level fixed effects. Fixed effects are omitted for parsimony. Standard errors are robust to heteroscedasticity and clustered at the firm level, following the suggestions in Petersen (2009). We perform one-sided t-tests on each coefficient. T-statistics are in parentheses. ***, **, and * denote significance at the 1, 5, and 10% level, respectively. Our control variables are defined in Appendix Table A1.

#### Change of Sample Period

In order to examine whether our basic results vary during the different lengths of the sample period, we change the time interval to discriminate policy sensitivity to time. By using mandatory CSR disclosure enacting in 2008 as a middle point, we restrict our sample to 3, 4, 5, and 6 years before and after 2008, respectively. The untabulated test reveals that the coefficients on *MANDATORY* remain positively significant, which proves that our basic results are robust.

#### Analysis of the Volatility of Tax Avoidance

A transparent information environment is correlated with high quality of governance, where firms tend to choose rather sustainable tax strategies (Neuman et al., 2013). In the front part of this article, we assume that the implementation of mandatory issuing CSR reports could increase firm transparency, thus we predict a smaller level of year-to-year volatility of tax avoidance. We use *StdDev_ETR* (*StdDev_NETR*) to measure firms’ volatility of tax avoidance, which is defined as the ratio of the standard deviation of annual *ETR*s (*NETR*s) to the absolute value of the mean of *ETR*s (*NETR*s) during the period from t–1 to t + 1 (Francis et al., 2019). Due to the missing data of nominal tax rate in 2018, we restrict our sample from 2002 to 2016. As shown in columns (3 and 4) of Table 4, *MANDATORY* is negatively significantly correlated with *StdDev_ETR* and *StdDev_NETR*, which proves a rather sustainable level of tax avoidance subsequent to the mandatory CSR disclosure because of a more transparent information environment.

#### Analysis of Ownership Type

Prior literature argued that agency costs in firms controlled by state entities are greater than other firms (Huang et al., 2011). Nearly half of the listed firms are politically connected through executives who are current or former government officials in China (Hung et al., 2012). As the controlling shareholder of state-owned firms, governments may mandate firms to accomplish political or social goals which arise agency costs between controlling shareholders and minority shareholders. Furthermore, executives in state-owned firms may realize private benefits by rent-seeking through CSR activities which results in a conflict between managers and shareholders (Chen et al., 2018). To sum up, state-owned firms face more political pressure compared with other firms, which may influence the negative correlation between mandatory CSR disclosure and tax avoidance.

In this section, we test whether our basic results are different between SOEs and Non-SOEs. *SOE* is defined as a firm that is ultimately controlled by governments. We separate the full sample into SOEs and non-SOEs and rerun the basic regression. Untabulated results reveal that the coefficients on *MANDATORY* are positively significant for SOEs but insignificant for non-SOEs using two measurements of tax avoidance. These results reveal that state-owned firms which are required to disclosure CSR reports tend to be less tax aggressive, while there is no difference in tax avoidance for other firms after mandatory CSR disclosure. It proves that state-owned firms indeed bear more political and social costs.

## Conclusion

This paper seeks to provide evidence on the effects of mandatory CSR disclosure on tax avoidance and tax incidence of various stakeholders. We find that firms tend to be less tax aggressive after the implementation of mandatory CSR disclosure since the end of 2008. We also extend prior research by providing evidence of whether the change of tax avoidance subsequent to the mandatory CSR disclosure has an effect on the tax incidence of kinds of stakeholders. It shows that consumers, suppliers, other stakeholders, employees, and shareholders bear the costs of the decrease in tax avoidance subsequent to mandatory CSR disclosure, while governments, banks, and other creditors do not bear the tax incidence. To make sure that our basic empirical results are robust, we first employ another two alternative measures of tax avoidance to prove that our main results are not restricted by specific measures. Then we change the span of the sample period to make sure that our basic results have no time sensitivity. Furthermore, we use the normalized standard deviation of *ETR* (*NETR*) to prove a rather sustainable level of tax avoidance subsequent to the mandatory CSR disclosure due to a more transparent information environment. Finally, we make additional analysis on whether our basic results differ for SOEs and non-SOEs.

Our study makes several contributions to both the corporate social responsibility and tax literature. First, the implementation of mandatory CSR disclosure since 2008 offers us a quasi-experiment setting to provide evidence that mandatory CSR disclosure affects the level of firms’ tax avoidance. Prior literature focuses on the benefits of voluntary CSR disclosure, however, we add more evidence from the perspective of tax avoidance in a mandatory disclosure environment. Second, our study supplements prior studies which document the influences of effective tax rate changes on firm stakeholders. While existing literature identifies incidence effects only on shareholders and employees, our study provides a more comprehensive examination of incidence effects on various kinds of stakeholders. Furthermore, compared with extant studies which use macro data, we use firm-level data from cash flow statements to identify incidence which could better portray mutual influence between firms and their various kinds of stakeholders. Future research could make a further test on whether firms’ characteristics or other factors mitigate or reinforce the incidence of each respective stakeholder.

## Data Availability Statement

Publicly available datasets were analyzed in this study. This data can be found here: https://www.gtarsc.com/.

## Author Contributions

RD and YS conceived and designed the study and draft the manuscript. RD contributed to the acquisition, analysis, and interpretation of data for the work. YS revised it critically and provided thoughtful and constructive suggestions. YC guided the research and made the final proofreading. All authors contributed to the article and approved the submitted version.

## Conflict of Interest

The authors declare that the research was conducted in the absence of any commercial or financial relationships that could be construed as a potential conflict of interest.

## Publisher’s Note

All claims expressed in this article are solely those of the authors and do not necessarily represent those of their affiliated organizations, or those of the publisher, the editors and the reviewers. Any product that may be evaluated in this article, or claim that may be made by its manufacturer, is not guaranteed or endorsed by the publisher.

## Appendix

**TABLE A1:** Variable definitions.

Variable	Definition
*ETR*	The ratio of total income tax expense paid to pre-tax profit, which is constrained to lie on the [–1,1] interval
*NETR*	The ratio of *ETR* to the nominal tax rate
*CETR*	The ratio of cash taxes paid to pre-tax profit, which is constrained to lie on the [–1,1] interval
*NCETR*	The ratio of *CETR* to the nominal tax rate
*StdDev_ETR*	The ratio of the standard deviation of annual *ETR*s to the absolute value of the mean of *ETR*s during the period from t–1 to t + 1
*StdDev_NETR*	The ratio of the standard deviation of annual *NETR*s to the absolute value of the mean of *NETR*s during the period from t–1 to t + 1
*MANDATORY*	A dummy variable equal to 1 if the firm is mandated to issue CSR reports in the current year, and 0 otherwise
*INCREASE1*	A dummy variable equal to 1 if the firm experience an increase in *ETR* in the current year, and 0 otherwise
*INCREASE2*	A dummy variable equal to 1 if the firm experience an increase in *NETR* in the current year, and 0 otherwise
*ROA*	Earnings before interest and tax divided by total assets
*SALES*	The natural log of sales
*LEV*	The ratio of long-term debt to total assets
*PPE*	The ratio of the sum of property, plant, and equipment to total assets
*INTANG*	The ratio of the net balance of intangible assets to total assets
*INVENTORY*	The ratio of the net balance of inventories to total assets
*SIZE*	The natural log of total assets
*MB*	Market-to-book ratio, calculated as the market value of equity divided by the book value of equity
*CONSUMER*	Cash received for selling goods and providing services divided by lagged total assets
*SUPPLIER*	Cash paid for purchasing goods and receiving services divided by lagged total assets
*GOVERN*	Cash payment of taxes and expenses divided by lagged total assets
*OSTAKE*	Other cash paid to relating operating activities divided by lagged total assets
*BANK*	Cash payment of borrowings divided by lagged total assets
*EMPLOYEE*	Cash paid to and on behalf of employees divided by lagged total assets
*SHAREHOLDER*	Cash paid for distributing dividends, profits, or interest payment divided by lagged total assets
*SOE*	A dummy variable equal to 1 if the firm is ultimately controlled by the government, and 0 otherwise

## References

[B1] Albertazzi U. Gambacorta L. (2010). Bank profitability and taxation. *J. Bank. Finance* 34 2801–2810. 10.1016/j.jbankfin.2010.06.003

[B2] Arulampalam W. Devereux M. P. Maffini G. (2012). The direct incidence of corporate income tax on wages. *Eur. Econ. Rev.* 56 1038–1054. 10.1016/j.euroecorev.2012.03.003

[B3] Azémar C. Hubbard R. G. (2015). Country characteristics and the incidence of capital income taxation on wages: an empirical assessment. *Can. J. Econ.* 48 1762–1802. 10.1111/caje.12179

[B4] Basov S. Bhatti I. (2016). “The incidence of taxation,” in *Islamic Finance in the Light of Modern Economic Theory*, eds Basov S. Bhatti I. (Berlin: Springer), 55–59. 10.1057/978-1-137-28662-8_4

[B5] Benlemlih M. Bitar M. (2018). Corporate social responsibility and investment efficiency. *J. Bus. Ethics* 148 647–671. 10.1007/s10551-016-3020-2

[B6] Blake D. R. (1979). Property tax incidence: an alternative view. *Land Econ.* 55 521–531. 10.2307/3145773

[B7] Carroll A. B. (1979). A Three-Dimensional Conceptual Model of Corporate Performance. *Acad. Manage. Rev.* 4 497–505. 10.5465/amr.1979.4498296

[B8] Chen S. Chen X. Cheng Q. Shevlin T. (2010). Are family firms more tax aggressive than non-family firms? *J. Financ. Econ.* 95 41–61. 10.1016/j.jfineco.2009.02.003

[B9] Chen Y.-C. Hung M. Wang Y. (2018). The effect of mandatory CSR disclosure on firm profitability and social externalities: evidence from China. *J. Account. Econ.* 65 169–190. 10.1016/j.jacceco.2017.11.009

[B10] Chiorazzo V. Milani C. (2011). The impact of taxation on bank profits: evidence from EU banks. *J. Bank. Finance* 35 3202–3212. 10.1016/j.jbankfin.2011.05.002

[B11] Chiou L. Muehlegger E. (2014). Consumer response to cigarette excise tax changes. *Natl. Tax J.* 67 621–650. 10.17310/ntj.2014.3.05

[B12] Cho S. Y. Lee C. Pfeiffer R. J. Jr. (2013). Corporate social responsibility performance and information asymmetry. *J. Account. Public Policy* 32 71–83. 10.1016/j.jaccpubpol.2012.10.005

[B13] Davis A. K. Guenther D. A. Krull L. K. Williams B. M. (2016). Do Socially Responsible Firms Pay More Taxes? *Account. Rev.* 91 47–68. 10.2308/accr-51224

[B14] Desai M. A. Dyck A. Zingales L. (2007). Theft and taxes. *J. Financ. Econ.* 84 591–623. 10.1016/j.jfineco.2006.05.005

[B15] Dhaliwal D. S. Li O. Z. Tsang A. Yang Y. G. (2011). Voluntary nonfinancial disclosure and the cost of equity capital: the initiation of corporate social responsibility reporting. *Account. Rev.* 86 59–100. 10.2308/accr.00000005

[B16] Dhaliwal D. S. Radhakrishnan S. Tsang A. Yang Y. G. (2012). Nonfinancial disclosure and analyst forecast accuracy: international evidence on corporate social responsibility disclosure. *Account. Rev.* 87 723–759. 10.2308/accr-10218

[B17] Dyreng S. D. Hanlon M. Maydew E. L. (2008). Long-run corporate tax avoidance. *Account. Rev.* 83 61–82. 10.2308/accr.2008.83.1.61

[B18] Edmans A. (2011). Does the stock market fully value intangibles? Employee satisfaction and equity prices. *J. Financ. Econ.* 101 621–640. 10.1016/j.jfineco.2011.03.021

[B19] Fankhauser S. Martin N. (2010). The economics of the CDM levy: revenue potential, tax incidence and distortionary effects. *Energy Policy* 38 357–363. 10.1016/j.enpol.2009.09.026

[B20] Francis J. R. Neuman S. S. Newton N. J. (2019). Does Tax Planning Affect Analysts’ Forecast Accuracy? *Contemp. Account. Res.* 36 2663–2694. 10.1111/1911-3846.12515

[B21] Frank M. M. Lynch L. J. Rego S. O. (2009). Tax reporting aggressiveness and its relation to aggressive financial reporting. *Account. Rev.* 84 467–496. 10.2308/accr.2009.84.2.467

[B22] Fuest C. Peichl A. Siegloch S. (2018). Do higher corporate taxes reduce wages? Micro evidence from Germany. *Am. Econ. Rev.* 108 393–418. 10.1257/aer.20130570

[B23] Fullerton D. Heutel G. (2007). Who bears the burden of a tax on carbon emissions in Japan? *Environ. Econ. Policy Stud.* 8 255–270. 10.1007/BF03353960

[B24] Fullerton D. Metcalf G. E. (2002). Tax incidence. *Handbook Public Econ.* 4 1787–1872.

[B25] Goolsbee A. (1998). Investment tax incentives, prices, and the supply of capital goods. *Q. J. Econ.* 113 121–148. 10.1162/003355398555540

[B26] Han X. Gong Q. Wu L. (2016). Payroll Tax Deduction and Corporation Payroll Arrangement. *Econ. Res. J.* 10 140–154.

[B27] Harberger A. C. (1962). The incidence of the corporation income tax. *J. Polit. Econ.* 70 215–240. 10.1086/258636

[B28] Harberger A. C. (2006). “Taxation and Income Distribution: Myths and Realities,” in *The Challenges of Tax Reform in a Global Economy*, eds Alm J. Martinez-Vazquez J. Rider M. (New York, NY: Springer).

[B29] Hassett K. A. Mathur A. (2015). A spatial model of corporate tax incidence. *Appl. Econ.* 47 1350–1365. 10.1080/00036846.2014.995367

[B30] Hoi C. K. Wu Q. Zhang H. (2013). Is corporate social responsibility (CSR) associated with tax avoidance? Evidence from irresponsible CSR activities. *Account. Rev.* 88 2025–2059. 10.2308/accr-50544

[B31] Huang W. Jiang F. Liu Z. Zhang M. (2011). Agency cost, top executives’ overconfidence, and investment-cash flow sensitivity—Evidence from listed companies in China. *Pacific Basin Finance J.* 19 261–277. 10.1016/j.pacfin.2010.12.001

[B32] Hung M. Wong T. J. Zhang T. (2012). Political considerations in the decision of Chinese SOEs to list in Hong Kong. *J. Account. Econ.* 53 435–449. 10.1016/j.jacceco.2011.10.001

[B33] Kim J. McGuire S. T. Savoy S. Wilson R. Caskey J. (2019). How Quickly Do Firms Adjust to Optimal Levels of Tax Avoidance? *Contemp. Account. Res.* 36 1824–1860. 10.1111/1911-3846.12481

[B34] Kitzmueller M. Shimshack J. (2012). Economic perspectives on corporate social responsibility. *J. Econ. Literat.* 50 51–84. 10.1257/jel.50.1.51

[B35] Kopczuk W. Marion J. Muehlegger E. Slemrod J. (2013). *Do the Laws of Tax Incidence Hold? Point of Collection and the Pass-through of State Diesel Taxes.* Cambridge, MA: National Bureau of Economic Research.

[B36] Kotlikoff L. J. Summers L. H. (1986). *Tax incidence.* (Cambridge, Mass: National Bureau of Economic Research).

[B37] Marion J. Muehlegger E. (2011). Fuel tax incidence and supply conditions. *J. Public Econ.* 95 1202–1212. 10.1016/j.jpubeco.2011.04.003

[B38] Mieszkowski P. (1972). The property tax: an excise tax or a profits tax? *J. Public Econ.* 1 73–96. 10.1016/0047-2727(72)90020-5

[B39] Murphy C. (2016). *The Effects on Consumer Welfare of a Corporate Tax Cut.* Australia: Crawford School of Public Policy, Australian National University.

[B40] Nerudová D. Dobranschi M. (2016). The impact of tax burden overshifting on the Pigovian taxation. *Procedia-Social Behav. Sci.* 220 302–311. 10.1016/j.sbspro.2016.05.503

[B41] Neuman S. S. Omer T. C. Shelley M. K. (2013). *Corporate Transparency, Sustainable Tax Strategies, and Uncertain Tax Activities.* Available online at: https://ssrn.com/abstract=2184892 (accessed December 31, 2021).

[B42] Pechman J. A. Okner B. A. (1974). *Who Bears the Tax Burden?.* Washington, D.C: Brookings Institution.

[B43] Petersen M. A. (2009). Estimating standard errors in finance panel data sets: comparing approaches. *Rev. Financ. Stud.* 22 435–480. 10.1093/rfs/hhn053

[B44] Rozema K. (2018). Tax Incidence in a Vertical Supply Chain: Evidence from Cigarette Wholesale Prices. *Natl. Tax J.* 71 427–450. 10.17310/ntj.2018.3.01

[B45] Servaes H. Tamayo A. (2013). The Impact of Corporate Social Responsibility on Firm Value: The Role of Customer Awareness. *Manage. Sci.* 59 1045–1061. 10.1287/mnsc.1120.1630 19642375

[B46] Suárez Serrato J. C. Zidar O. (2016). Who benefits from state corporate tax cuts? A local labor markets approach with heterogeneous firms. *Am. Econ. Rev.* 106 2582–2624. 10.1257/aer.20141702

[B47] West S. E. Williams R. C. (2004). Estimates from a consumer demand system: implications for the incidence of environmental taxes. *J. Environ. Econ. Manage.* 47 535–558.

